# Immunological Aspects of Chronic Rhinosinusitis

**DOI:** 10.3390/diagnostics12102361

**Published:** 2022-09-29

**Authors:** Katarzyna Czerwaty, Katarzyna Piszczatowska, Jacek Brzost, Nils Ludwig, Mirosław J. Szczepański, Karolina Dżaman

**Affiliations:** 1 Department of Otolaryngology, The Medical Centre of Postgraduate Education, 01-813 Warsaw, Poland; 2 Department of Biochemistry, Medical University of Warsaw, 02-097 Warsaw, Poland; 3 The Children’s Memorial Health Institute, 04-730 Warsaw, Poland; 4 Department of Oral and Maxillofacial Surgery, University Hospital Regensburg, 93053 Regensburg, Germany

**Keywords:** chronic rhinosinusitis, immunology, inflammation, sinusitis, nasal polyps, inflammatory endotype

## Abstract

Chronic rhinosinusitis (CRS) is related to persistent inflammation with a dysfunctional relationship between environmental agents and the host immune system. Disturbances in the functioning of the sinus mucosa lead to common clinical symptoms. The major processes involved in the pathogenesis of CRS include airway epithelial dysfunctions that are influenced by external and host-derived factors which activate multiple immunological mechanisms. The molecular bases for CRS remain unclear, although some factors commonly correspond to the disease: bacterial, fungal and viral infections, comorbidity diseases, genetic dysfunctions, and immunodeficiency. Additionally, air pollution leads increased severity of symptoms. CRS is a heterogeneous group of sinus diseases with different clinical courses and response to treatment. Immunological pathways vary depending on the endotype or genotype of the patient. The recent knowledge expansion into mechanisms underlying the pathogenesis of CRS is leading to a steadily increasing significance of precision medicine in the treatment of CRS. The purpose of this review is to summarize the current state of knowledge regarding the immunological aspects of CRS, which are essential for ensuring more effective treatment strategies.

## 1. Introduction

**Figure 1:**
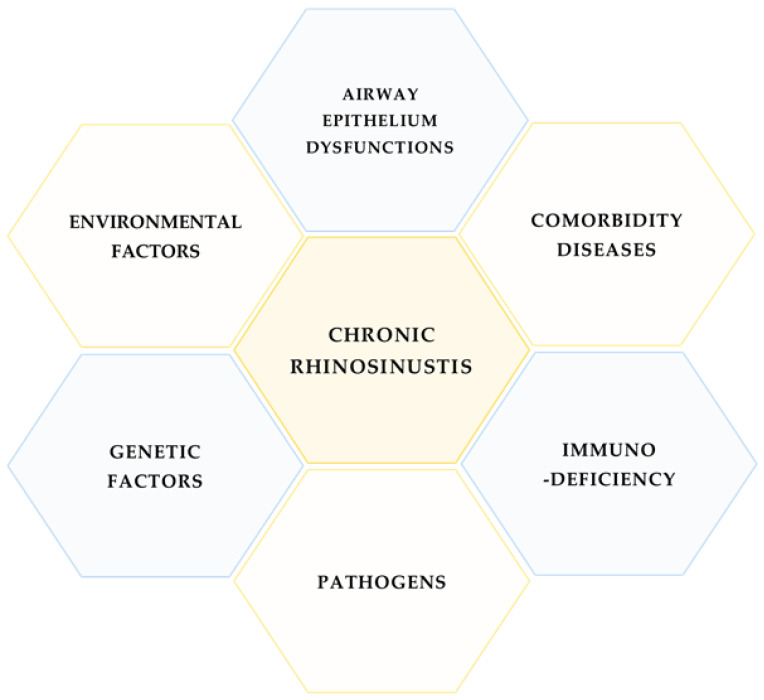
Main factors related to CRS.

Chronic rhinosinusitis (CRS) is a heterogeneous group of sinus diseases with unclear molecular bases, although some factors are associated with the disease: bacterial, fungal and viral infections, comorbidity diseases, genetic dysfunctions, and immunodeficiency (Figure 1).

**Figure 2:**
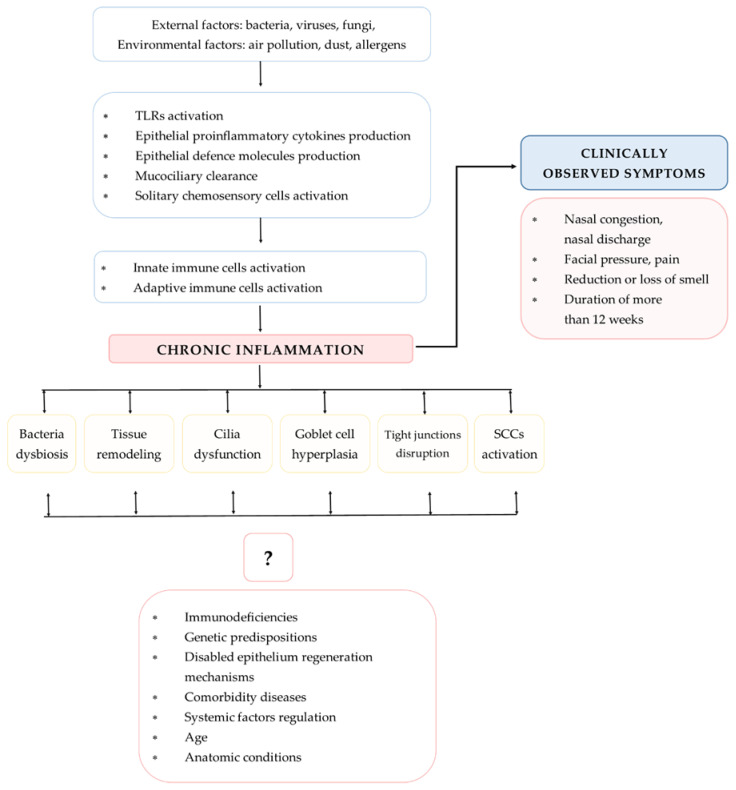
Mechanisms involved in CRS pathogenesis (TLRs—Toll-like receptors; SCCs—solitary chemosensory cells).

The different types of CRS reflect the variety of immunological response pathways and advancements in the understanding of the immunology and endotyping of CRS that are essential for improving the treatment regimen. The major processes involved in the pathogenesis of CRS include airway epithelium (AE) dysfunctions that are influenced by external and host-derived factors. The pathogenesis is mainly influenced by the activation of multiple immunological mechanisms, leading to persistent chronic inflammation (PCI). The purpose of this review is to summarize selected mechanisms of CRS pathogenesis with particular attention to immunological aspects of the disease (Figure 2).

## 2. Immunological Response Pathways in CRS

CRS and PCI are both characterized by a dysfunctional relationship between environmental factors (EFs) and the host immune system. Immune response pathways depend on various factors including the endotype and genotype of the patient; however, the detailed mechanisms that mediate CRS immunopathogenesis are complex and still not sufficiently explained.

CRS can be divided into CRS with nasal polyps (CRSwNP) and chronic rhinosinusitis without nasal polyps (CRSsNP) depending on the endoscopically observed presence of nasal polyps in the middle nasal meatus. Classification that better reflects underlying pathomechanisms is based on endotypes [1]. Three main inflammatory endotypes, type 1, type 2 and type 3, are controlled by distinct gene signatures and can be found in both CRSwNP and CRSsNP phenotypes [2,3]. In Western countries, CRSwNP is mainly associated with type 2 inflammation [1].

**Figure 3:**
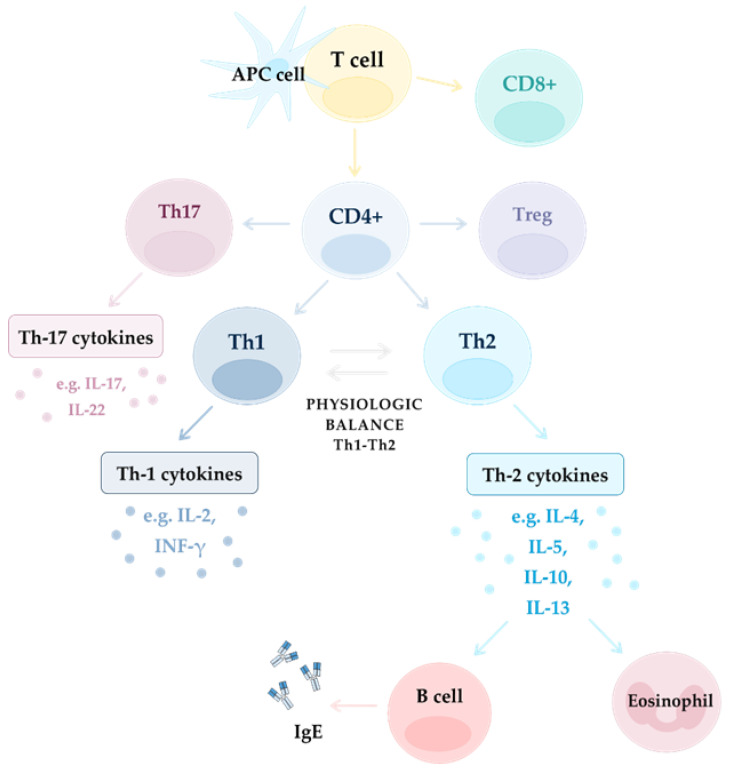
Mechanism of inflammation in different endotypes: Th-2 dependent and non-Th-2 dependent (Th1 and Th17 dependent).

The sinus mucosa (SM) is an anatomical site that is exposed to EFs and where precisely regulated cooperation between innate and adaptive immunity is crucial for homeostatic balance. In CRS, mucosa activity is compromised by PCI, leading to tissue remodeling, which might result from AE cells dysfunction and enhanced barrier permeability [1]. The first line of defense in SM constitutes the mechanical barrier of AE and mucociliary clearance. The next line of local defense provides the innate and complement immune system (IIS and CIS, respectively). Their activities lead to the identification and elimination of external pathogenic agents and also antigen presentation to activate cells of the adaptive immune response (AIR) [1]. The AIR appears later, but is highly specialized in action and employs multiple subpopulations of T and B cells (Figure 3).

### 2.1. Airway Epithelium as the First Line of Defense in Sinuses

#### 2.1.1. Structure and Functions of Sinusal Airway Epithelium

Mucociliary clearance is the primary innate defense mechanism, supporting mucous relocation and removal. Mucus is mainly produced by goblet cells (GCs) and contains mucins with antibacterial properties. Impairment of AE cilia functions in CRS impedes mucous transport and elimination. A decrease in the differentiation of ciliated cells and beating frequency is mediated by interferon-γ (IFN-γ) and interleukin-13 (IL-13) and is also noticeable in GC hyperplasia [4]. Primary ciliary dyskinesia may also be the reason for mucociliary clearance disabilities [1]. Additionally, the eosinophilic endotype of CRSwNP is characterized by increased levels of two main airway mucins that are involved in mucous formulation (MUC5AC and MUC5B) and of the anion exchanger pendrin [5,6,7]. Increased MUC5AC expression and GC metaplasia were also observed in human CRS sinonasal tissue in response to cigarette smoke exposure [8]. In addition, several multifunctional proteins are upregulated in the mucus of CRSwNP such as cystatin 2, pappalysin-A, periostin, and serpins. Periostin expression is associated with the presence of basement membrane thickening (BMT), fibrosis, and tissue eosinophilia [9] and may be involved in the remodeling of NPs [10]. Cystatin 2 triggers epithelial barrier functions and immunomodulation. Cystatin SN, a type 2 cysteine protease inhibitor, was increased in patients with eosinophilic CRSwNP but decreased in patients with non-eosinophilic CRSwNP in comparison to control subjects [11]. High levels of cystatin SN in nasal secretions are correlated with a faster onset and higher rate of uncontrolled status in CRSwNP [12]. Pappalysin-A stimulates proliferation mediated by the insulin-like growth factor 1. Periostin leads to proliferation, angiogenesis, invasion, eosinophil recruitment, Th2 immune response, and airway remodeling. Serpins, especially serpinF2 and serpinE1, trigger the inhibition of fibrinolysis. Interestingly, after surgery, levels of cystatin 2, pappalysin A, periostin, and serpinF2 decrease, whereas serpinE1 increases, and in the course of the follow-up period—levels of cystatin 2, pappalysin A, and periostin increase [13]. Proteomic analysis of SM samples from CRS patients demonstrated downregulation of pathways associated with mucosal immunity and upregulation of cellular metabolism related to tissue remodeling [14]. Mucus inflammatory proteins might be also involved in olfaction dysfunction and their profile is distinct when comparing CRSwNP and CRSsNP patients [15]. Tight junctions (TJs) by closely located AE cells form selectively permeable barriers. In the course of CRS, levels of molecules building TJs decrease: JAM-A, E-cadherin [16], zonula occludens 1 (ZO-1), occludin 1 [17], and also IFN-γ and IL-4, which most likely play an important role in this process [18]. Barrier integrity and cilia dysfunction are also mediated by a decrease in p63 [19], as well as decreased Wnt pathway activity that may lead to NP formation by reprogramming the epithelium morphology, especially cilium and adherens junctions [20].

#### 2.1.2. Secretory Functions of Airway Epithelium

**Figure 4:**
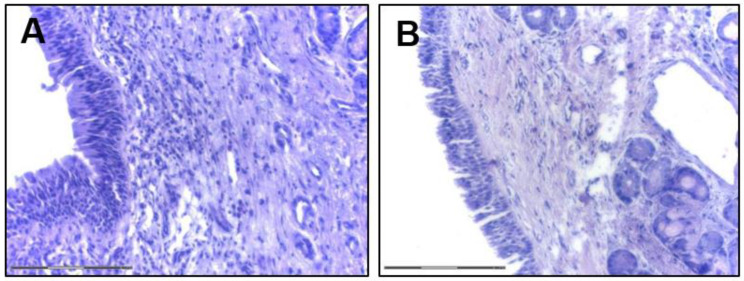
Sinusal airway epithelium (H and E staining, bar indicates 150 μm; courtesy of K. Piszczatowska). (**A**) CRSwNP; (**B**) CRSsNP.

Another function of the AE is the secretion of defense molecules acting against pathogens. The AE secretome includes lysozyme, lactoferrin, hydrogen peroxide, and nitric oxide (NO) and in the course of CRS secretion of dual oxidases 1 and 2 (DUOX1 and DUOX2, respectively) is accelerated and responsible for hydrogen peroxide production [21]. In response to stimulation with external pathogens and EFs the epithelium releases cytokines and in CRS, IL-25, IL-33, TSLP (thymic stromal lymphopoietin) play a crucial role. Additionally, those cytokines are released by immune cells—IL-33 is mainly produced by macrophages and dendritic cells (DCs) [22] and IL-25 is produced by eosinophils or mast cells (MCs) [23,24]. IL-25 interacts with nasal fibroblasts [25] that are possibly involved in NP formation [24,26]. Similarly, IL-25 triggers lung fibrosis by reprogramming alveolar epithelial cells and fibroblasts [27]. Experimental studies showed that cells stimulated with poly (I:C) release IL-25 and TSLP [28] and that overexpression of TSLP correlates with induction of Th2 inflammatory factors [29]. IL-33 modulates Th2 cytokine production [30] and upregulated levels of IL-33 in the NPs might also contribute to mucosal repair functions by activating the Notch-1 signaling pathway [31]. In the CRSwNP, TSLP and IL-33 activate ILC2 to produce IL-5 that activates eosinophils together with IL-13 [32]. MCs, especially in the eosinophilic CRSwNP, produce periostin [33] and periostin stimulates the secretion of TSLP by the epithelium which activates MCs to produce IL-5, ultimately stimulating DCs. Those may lead to Th2 response and eosinophilia in CRSwNP [34] (Figure 4).

#### 2.1.3. Chemosensory Cells in the Pathogenesis of CRS

Solitary chemosensory cells (SCCs) are present in the AE [35] and appear widely in NPs. They are efficient producers of IL-25 which activates ILC2 responsible for mediation of the Th2 immune response pathway [36,37]. The surface of SSCs is decorated by many forms of taste receptors (T2Rs) with immunoregulatory functions. Bitter stimulation of T2Rs leads to antimicrobial peptide and NO production, as well as elevated ciliary beating frequency. Hereby, the functions of T2Rs in the upper respiratory epithelium may be genetically dependent [38]. In the case of CRSsNP the non-tasting genotype of a bitter taste receptor, taste receptor 2 member 38 (T2R38), relates to increased Gram-negative bacteria colonization and a worse course of disease [39]. Interestingly, the human T2R38 can be detected on the surface of some immune cell populations, for instance on CD4+ and CD8+ T lymphocytes, and is stronger pronounced in lymphocytes of younger than elderly patients [40]. In addition, T2Rs present in SM contribute to NO production which improves its defense properties [41]. T2Rs also occur on lung macrophages and stimulation with receptor agonists resulted in a decrease in cytokine production [42].

#### 2.1.4. TLRs in the Pathogenesis of CRS

TLRs (Toll-like receptors) are present on the cell surface and also in endosomes, endoplasmic reticulum, and lysosomes and recognize pathogen associated molecular patterns (PAMPs). Depending on their cellular localization they identify pathogen membrane surfaces or nucleic acids [43,44]. Activation of AE-associated TLRs by pathogens triggers the production and release of cytokines, chemokines, and defense molecules and the activation of immune cells, thus, TLRs contribute to initiating and maintaining an inflammatory response. TLRs can also activate interferon I after exposure to viruses [45]. In the case of CRSsNP, TLR2, TLR4, transforming growth factor β (TGFβ), and collagen are upregulated in comparison to CRSwNP and expression of TLR2 and TLR4 correlate with neutrophil infiltration [46]. In the case of CRSwNP upregulation of TLR2 leads to Th17/T regulatory cell (Treg) imbalance and treatment of peripheral blood mononuclear cells with *Aspergillus flavus* accelerates T17-mediated inflammation [47]. Additionally, overexpression of TLR2 and nuclear factor κβ (NF-κβ) in the CRS mucosa is associated with biofilm formation [48].

#### 2.1.5. Hypoxic Conditions in the Airway Epithelium

Hypoxic conditions contribute to immune regulation in CRSwNP by triggering an increase in levels of IL-17A, hypoxia-inducible factor 1α (HIF-1α), and HIF-2α [49]. HIF-1α expression in SM of patients with CRSwNP is significantly increased compared to SM of healthy controls and the HIF-1α level in polyp tissues is positively associated with IL-17A production and neutrophilic inflammation [50]. Experimental in vitro models of nasal epithelial cells cultured under hypoxic conditions showed intensified chemokine secretion and chemotaxis of eosinophils and neutrophils compared to normoxic conditions [51] and increased levels of Eotaxin-1 (CCL11), Eotaxin-2 (CCL24), and Eotaxin-3 (CCL26) in NPs [52]. Another study demonstrated that hypoxic conditions that may appear in mucosa during sinusitis lead to increased HIF-1α and additionally MUC5AC expression [53].

### 2.2. Innate Immune Cell Response in CRS

**Figure 5:**
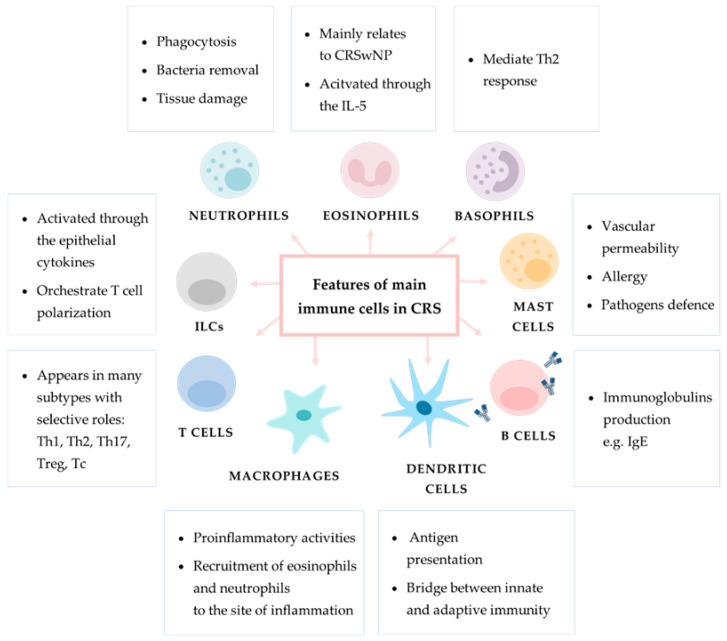
General information regarding the role of main immune cells in chronic inflammation in CRS. In the boxes provided are descriptions of particular immune cells’ main functions.

General information regarding the role of immune cell populations in chronic inflammation in CRS are shown in Figure 5.

#### 2.2.1. Innate Lymphoid Cells

The main activators of innate lymphoid cells (ILCs) are the epithelial cytokines IL-25, IL-33, and TSLP. Activated ILCs produce the proinflammatory cytokines IFN-γ, IL-5, IL-13, IL-22, and IL-17A [54] and modulate functional responses of other immune cell populations. ILC1, ILC2, and ILC3 cooperate, respectively, with the CD4+ T lymphocyte subsets Th1, Th2, and Th17 [55]. Each ILC type produces proper cytokines albeit are able to overtake the function of others depending on stimulation with epithelial cytokines or antigen-presenting cells [56]. In general, ILC1 regulates response to viruses and intracellular bacteria and promotes Th1 response with secretion of cytokines, mainly IFN-γ. ILC2 are responsible for responding to parasites, allergy, and trigger of tissue repair and favor a type 2 response orchestrated by the cytokines IL-4, IL-5, and IL-13. ILC3 corresponds to extracellular organisms, relates to Th17 immune response, and secretes IL-17 and IL-22 [57,58]. To the best of current knowledge, the main role in CRS play ILC2s. However, ILC1s and ILC3s also appear in CRS, but their functional contribution remains unclear.

#### 2.2.2. Neutrophils

Neutrophils are mainly activated by microbes, tissue damage, epithelial IL-8 or fungi and contribute to phagocytosis as well as incapacitation of extracellular microbes. The role of neutrophils in CRS remains unclear; however, they are associated with CRSwNP in Asia, significantly more when comparing to the Caucasian population [59]. Neutrophils contribute to tissue damage and barrier disruption by degranulation [60,61], but on the other hand can secrete oncostatin M that triggers repair of epithelial functions and integrity [62] and counteracts the profibrotic effect of IL-4 and TGFβ1 [63]. The polyp tissue microenvironment leads to the differentiation of IL-17-positive T cells and their quantity correlates with infiltration of neutrophils. Additionally, it has been reported that *Staphylococcus aureus* (*SA*) might be involved in upregulation of IL-17- and IL-17-positive T cells in NPs [64]. The amount of neutrophil extracellular traps (NETs) in nasal secretion, which participate in innate immunity by trapping microorganisms, is increased in exacerbated CRS in comparison to stable CRS [65]. NETs are significantly increased in NPs which indicates a potential role in pathogenesis of neutrophil inflammation in CRSwNP [66].

#### 2.2.3. Monocytes and Macrophages

Monocytes play a role in the elimination of microbes from the blood and tissues. In the site of inflammation monocytes are able to transfer into macrophages which differentiate into two distinct phenotypes: M1 macrophages which are active in early inflammatory processes-promoted by T1 cytokines and M2 macrophages stimulated by Th2 cytokines. Studies have shown that M2 macrophages in NPs may also be involved in fibrin deposition modulated by the factor XIII-A. Macrophages attract neutrophils and eosinophils to the inflammatory site [67,68,69]. In NP tissue, M1 macrophages are the major cellular source of IL-17A and a possible influence on NP formation was demonstrated in a murine NP model [70].

#### 2.2.4. Basophils

Basophils mostly circulate in the blood; however, their release of IL-4 triggers a Th2-mediated inflammatory response [71]. Their increased levels were detected in NPs of patients with aspirin exacerbated respiratory disease (AERD) compared to CRSwNP patients, which may contribute to severity unique to AERD [72]. The role of basophils in CRS immunopathology remains unclear and needs further investigation.

#### 2.2.5. Mast Cells

MCs occur in connective tissues, under the epithelium and in the neighborhood of glandular tissue inside polyps and are activated by stimulation of TLRs with microbes, CIS or antibodies. Degranulation of MC components leads to increased vascular permeability, pathogen defense, allergy and finally, tissue oedema, extracellular matrix (ECM) degradation, and disabled epithelial barrier integrity [73,74,75,76,77]. In the case of CRS MCs contribute to eosinophilic inflammation [78] and trigger CRSwNP and AERD through the release of leukotrienes (cysLTs) or prostaglandins (PGD2) [79,80]. MCs also release periostin that is a meaningful factor with regard to the eosinophilic CRSwNP [33].

#### 2.2.6. Eosinophils

Eosinophilic inflammation is more prevalent with regard to CRSwNP [81]; however, it negatively impacts the course of the disease, independent of the presence of NPs [82,83]. Especially recurrent CRSwNP patients have more eosinophil as well as mucin eosinophilic aggregates [84]. Feng et al. also indicated increased levels of eosinophils in the peripheral blood of eosinophilic CRS patients, suggesting its potential diagnostic value in evaluation of disease severity [85]. In CRS, the activation of eosinophils is maintained by epithelial cytokines, Th2 cytokines, proteases, components of the complement system, stem cell factors, and eicosanoids [86,87,88]; however, the biggest contributors are ILCs and Th2 cells [32]. Moreover, some microRNAs (miRNAs) might regulate eosinophil activity such as miR-125b that is enriched in eosinophilic CRSwNP [89]. Additionally, the elevated levels of IgE correlate with eosinophil infiltration and possibly lead to NP development [90] as well as Semaphorin 7A—a factor relevant to fibrinolysis that occurs on the airway eosinophils [91]. It was shown for CRSsNP patients with a total IgE serum concentration over 100 IU/mL that systemic steroid therapy is more effective than intranasal steroids [92].

#### 2.2.7. Natural Killer Cells

Natural killer cells (NKs) are cytotoxic lymphocytes with abilities to recognize and kill infected cells and release IFN-γ to stimulate macrophage activation [55]. In CRSwNP, NK cells have decreased degranulation properties and also IFN-γ or tumor necrosis factor α (TNF-α) production [93].

### 2.3. Adaptive Immune Response Cells in CRS

#### 2.3.1. Dendritic Cells

DCs present antigens to naïve T cells and in this way connect innate and AIR. Epithelial cytokines and ILCs activate DCs, leading to pertinent T cell polarization [55,94]. DCs might infiltrate NP tissue and activate T cells by the CD40/CD40L costimulatory molecules [94]. Other studies described increased levels of programmed cell death 1 (PD-1) in CRSwNP [95] and also programmed cell death 1 ligand 1 (PD-L1) in the case of eosinophilic endotype in Asia population [96]. In the eosinophilic CRSwNP, DCs expressing OX40 ligand (OX40L)/PD-L1 lead to activation of the Th1/Th2/Th17 pathway, whereas in non-eosinophilic CRSwNP, DCs with lower expression of OX40L/PD-L1 mediate Th1/Th17 response [97]. DCs isolated from CRS patients overexpress miR-150-5p that together with its target—early growth response 2 (EGR2)—trigger T cell activation and proliferation [98].

#### 2.3.2. T Cells

T cells have a variety of biological functions including effector cell recruiting, neutralization of infected cells, cooperation with B cells resulting in production of immunoglobulins, and their role as memory cells in IIC. The main subtypes of T cells are CD4+ T helper cells and CD8+ cytotoxic T cells. CD4+ T cells differentiate into five main subsets: Th1, Th2, Th17, follicular helper T cells, and Tregs [55]. Th1 cells are activated by phagocytosed microbes also with the support of ILC1s. Th1 cells release IFN-γ, TNF-α, and TNF-β that help in microbiome phagocytosis by activating macrophages and antigen presentation, as well as stimulating IgG production by B cells, neutrophils and local tissue inflammation [99,100]. Th2 are mainly activated by parasites which mobilize eosinophils, MCs, ILC2s and enhance the production of IgE [101]. Th2 cells release IL-4, IL-5, and IL-13 which contribute to activation of eosinophils, mucus production, and stimulation of macrophages which may produce growth factors, leading to tissue repair mechanisms [102]. The Th17 subpopulation activates neutrophils, monocytes and secretes IL-17A, IL-17F, and IL-22 [1].

The levels of proper T cell subtypes in CRS differ depending on the endotype and genotype. Increased levels of the Th2 subset with eosinophils appear in CRSwNP patients from Western regions and increased levels of Th1/Th17 and neutrophils are found in Asian population and additionally in patients with NPs [103,104,105]. Studies have shown a similar inflammatory profile of NPs and neighboring non-polypoidal mucosa from the same patients. NP tissues treated with *SA* enterotoxin B (SEB) are characterized by an activated Th2/Th17 response pathway in comparison to controls. In the NP tissue and non-polypoid tissue in comparison to control, gene expression levels of IL-5, IL-8, and TLR4 are increased and TBX21 (encodes for T-bet), FOXP3, IL-1B and IL-6 decreased. It was demonstrated that treatment of NPs with SEB results in an increase in gene expression levels of IL-5 and IL-17A in the tissue and increased TLR4 and decreased IL-1B gene and protein levels in supernatants. Proinflammatory phenotype of contiguous tissue and decreased levels of antibacterial cytokines might be involved in disabled response to pathogens, chronic inflammation and also NP formation [106]. CD8+ T cells may transform into cytotoxic T cells with abilities to kill infected or damaged cells when stimulated by antigens or other factors [55]. It was demonstrated that higher levels of CD8+ than CD4+ T cells can be found in CRSwNP and that the local microenvironment of NPs promotes T cell variation [107] and the release of proinflammatory cytokines with antiapoptotic properties for T cells [108]. CD8+ T cells are upregulated in both: eosinophilic and non-eosinophilic CRSwNP [109].

Overall, in CRSsNP the Th1 and Th17 immune response pathways are activated and relate to the expression of TGFβ, INFs and IL-6, IL-8, and IL-17 [110]. In contrast, another analysis did not demonstrate significant differences in the level of IFN-γ between CRSwNP, CRSsNP and in comparison to the control group [111]. CD4+ Tregs characterized by Foxp3 expression have immunosuppressive functions. They are involved in self-antigen recognition, self-tolerance, and general homeostasis [1]. In CRS, decreased levels of Tregs may lead to a chronic inflammatory state [112] and overall, their levels are decreased in peripheral blood of CRS patients. The quantities of Tregs are similar in CRSwNP and CRSsNP patients, but the tissue infiltration levels of Tregs are higher in CRSwNP compared to CRSsNP. In CRS, Tregs express more proinflammatory than regulatory capacities [113] and additionally in CRSwNP the migration potential of Tregs is limited [114].

#### 2.3.3. B Cells

Activated B cells produce highly specialized immunoglobulins that bind antigens and play a crucial role in neutralization of many pathogenic factors. B cell levels are increased in the group of CRSwNP patients when compared to CRSsNP patients [115] and have an accelerated memory phenotype and are less mature or regulatory for Th lymphocytes [116]. Several factors are upregulated in CRS which have the potential to modulate B cell response pathways. The chemokines CXCL13 and CXCL12 are upregulated in CRS and lead to B cell recruitment [117]. IL-21, which is overexpressed in CRSwNP, activates B cell differentiation and leads to IgG and IgA secretion [118]. In CRSwNP, ILC2 triggers local B cell activation [119]. In the NP tissue, the close cooperation of B cells with MCs enhances local IgE production [77]. Additionally, in CRSwNP, activation of TLR9 leads to the release of type I interferon, ultimately increasing levels of B cell-activating factor [120]. The increased levels of B cell-activating factor in the serum positively correlate with blood eosinophil counts and percentages, tissue eosinophil counts, and total IgE in serum. Hereby, the levels of B cell-activating factor are significantly higher in patients with recurrent polyps, which might suggest its role in distinguishing CRSwNP endotypes and predicting postoperative recurrence [121].

## 3. Other Selected Aspects of CRS Pathogenesis

### 3.1. High Mobility Group Box (HMGB1) Protein and a Receptor for Advanced Glycation and Products (RAGE) Pathway in CRS

HMGB1 is an alarmin protein involved in many chronic inflammatory diseases and also plays a role in CRS pathogenesis. Tissue expression of HMGB1 and its receptor RAGE correlates with the disease course of CRSwNP. We have previously described that RAGE is more pronounced during disease development [122]. Similarly, we have also demonstrated that in the CRSsNP, HMGB1 expression in the tissue indicates no differences in comparison to healthy volunteers, meanwhile, RAGE is overexpressed, relates with disease activity and allergy [123]. Elevated levels of HMGB1 appear especially in eosinophilic CRSwNP [124]. Additionally, TLR4 is upregulated in CRSwNP tissue and is another HMGB1 receptor [125]. In CRSwNP, HMGB1 is overexpressed in the nucleus of epithelial cells, but decreased in cytoplasm [126], albeit hypoxic conditions regulate the functions of HMGB1 in the upper airway, triggering its translocation. HMGB1 leads also to the production of reactive oxygen species (ROS) in AE cells and ROS derived from DUOX2 leads to increased IL-8 secretion [127]. Interestingly, elevated levels of ROS in the freshly wounded nasal epithelial cells and fibroblasts obtained from CRS individuals might be reduced by some antibiotics and trigger a decline in nasal fibroblast migratory capacity without affecting nasal epithelial cells [128]. Recently, studies demonstrated the role of HMGB1-RAGE signaling pathway in the process of epithelial to mesenchymal transition (EMT) in the case of CRSwNP patients. Vetuschi et al. observed in CRSwNP tissues an upregulation of the AGE/RAGE/p-ERK pathway and also of the mesenchymal markers vimentin and IL-6, suggesting that their cooperation might be associated with tissue remodeling. However, the authors were not able to find any differences in the TGFβ/Smad3 pathway between CRSwNP and normal controls [129]. Similarly, another study demonstrated that HMGB1 promotes upregulation of mesenchymal markers (vimentin, α-SMA) and diminished epithelial markers (occludin, ZO-1, E-cadherin) and hypoxia—induced HMGB1 release might lead to EMT through the RAGE pathway [130]. In accordance with the findings of Vetuschi et al., HMGB1-treated cells did not secrete TGFβ on the apical and basal side, suggesting that EMT in CRSwNP might be TGFβ independent. Increased levels of HMGB1 appeared in cytoplasm or extranuclear compartments and NPs fluid in comparison to control mucosa. Additionally, HMGB1 instilled to the mouse model, proved their EMT inducing capacity and also analysis of human NP tissue indicated increased levels of HMGB1 as well as mesenchymal markers, whereas epithelial markers were decreased [130]. HMGB1 induced myofibroblast differentiation and ECM production in nasal fibroblast [131]. In the case of eosinophilic CRSwNP, when cells were treated with rhHMGB1 (recombinant human HMGB1), the expression of vimentin and N-cadherin was increased and the expression of E-cadherin and ZO-1 were decreased, both in a concentration-dependent manner. Use of peroxisome proliferator-activated receptor γ (PPAR-γ) agonist resulted in a decrease in lipopolysaccharide (LPS)-stimulated HMGB1 secretion and an EMT retraction [132].

### 3.2. Tissue Remodeling in CRS

**Figure 6:**
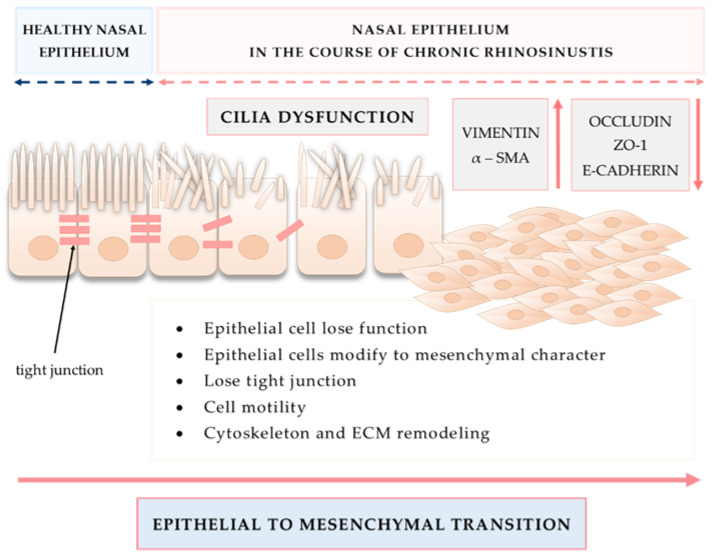
Epithelial to mesenchymal transition as an important mechanism involved in tissue remodeling in CRS.

An important mechanism involved in tissue remodeling is EMT, also presented in Figure 6.

During EMT, epithelial cells lose their functions and acquire the character of mesenchymal cells. Cells lose TJs, gain motility function, remodel cytoskeleton and ECM, and have altered gene and protein expression [133]. The TGFβ pathway is among the best known factors involved in the process of EMT. In eosinophilic and non-eosinophilic CRSwNP, miR-182 regulated EMT in response to TGFβ1 and might promote nasal polypogenesis [134]. The role of TGFβ1 is significantly more pronounced in CRSsNP than in the case of CRSwNP. In CRSsNP, TGFβ1 activates heat shock protein 47 (HSP47) that is overexpressed in the nasal fibroblasts and it leads to myofibroblast differentiation as well as ECM production. TGFβ1 induces HSP47 expression via the Smad 2/3 pathway. Interestingly, glucocorticosteroids were able to decline effects of HSP47 induction by TGFβ1 [135]. Additionally, the TGFβ/Smad 2/3 pathway leads to collagen and connective tissue growth factor (CTGF) production in fibroblasts obtained from CRSsNP mucosa [136]. The expression of relaxin-2, Smad2, Smad3 and TGFβ1 mRNA in the CRSsNP group was significantly higher than in the CRSwNP and control groups [137]. Moreover, miR-21 might be involved in EMT through the activation of the TGFβ1-mediated PTEN/Akt pathway [138].

Recently, it has been demonstrated that the cold-inducible RNA binding protein (CIRP) is upregulated in nasal epithelial cells from eosinophilic and non-eosinophilic CRS and macrophages. CIRP might contribute to edema formation through the capacity to stimulate metalloproteinase (MMP) and vascular endothelial growth factor A (VEGF-A) from nasal epithelial cells and macrophages [139]. Another study suggested the role of the PI3K/Akt/HIF-1α pathway in the inflammation and tissue remodeling in CRS. Nasal epithelial cells stimulated with LPS release IL-25, IL-17RB, HIF-1α and p-Akt. Levels of IL-25, IL-17RB, HIF-1α decrease after implementing the PI3K inhibitor [140]. Similarly, nasal fibroblasts obtained from CRS patients cultured in vitro, stimulated with LPS secrete TSLP in a TLR4-dependent manner and activate mitogen-activated protein kinase (MAPK), Akt, NF-κβ pathways. Additionally, use of ex vivo organ culture of nasal inferior turbinate model validated these results. Macrolides and corticosteroids were able to reduce expression of TSLP in the fibroblasts and downstream pathways [141]. The exposure of nasal fibroblasts on cigarette smoke exposure results in increased MMP-2 expression, inhibited tissue inhibitor of metalloproteinase-2 (TIMP-2) expression and induced ROS production [142].

Nasal fibroblasts play a crucial role in tissue remodeling. Nasal fibroblasts influenced by many factors secrete various signaling molecules and are able to differentiate into myofibroblasts. Hypoxic conditions trigger the differentiation of NP-derived fibroblasts into myofibroblasts in the manner dependent on ROS generated through the NOX4 (NADPH oxidase 4) and TGFβ1 [143]. Moreover, NOX1 and NOX4 are overexpressed in the mucosa of NPs-in the case of allergic rhinitis at the mRNA and protein levels [144]. Other studies described elevated NADPH oxidase subunits on the NP tissue, suggesting their role and also oxidative stress involvement in CRSwNP pathogenesis [145]. NP tissue has additionally decreased stem cell potential in comparison to healthy tissue from the same CRS patients, which is evaluated through the differentiation of isolated mesenchymal cells into adipocytes and it may lead to abate epithelium regeneration [146].

### 3.3. Neuro-Inflammation as an Emerging New Aspect of Airway Inflammatory Diseases

Airway epithelium anatomically and functionally collaborates with the nervous system. Various inflammatory mediators and irritants that appear in the area of the airway might stimulate nerves, leading to neurogenic inflammation. On the other hand, inflammation may also be enhanced through the neurotransmitters secreted by neurons and neuropeptides released from immune cells and non-neural cells during the disease state. Additionally, activation of SCCs present in the nasal epithelium might be involved in the neurogenic inflammatory pathway. The role of neuro-immune regulation in inflammation has been already the subject of research in airway inflammatory diseases, such as COPD and asthma [147,148,149].

It was also demonstrated that autonomic dysfunction symptoms significantly positively correlate with CRS severity, especially in a group of CRSwNP patients [150]. Histological examination showed abundant sympathetic fibers in the pedicle of NPs, but a lack of this innervation in the body and apex of the polyps, which can be important in the pathogenesis of NPs [151]. There is a hypothesis that the cholinergic system can be implicated in the inflammation of CRS, especially in CRSwNP [152]. It was observed that higher preoperative autonomic symptom scores corresponded to uncontrolled inflammation following functional endoscopic sinus surgery and symptoms of autonomic nervous system dysfunction improved following sinus surgery [153]. Chronic inflammation may also trigger neuron death mediated by c-Jun N-terminal kinases (JNK), leading to loss of olfaction, which is a common symptom in CRS [154].

### 3.4. Small Extracellular Vesicles (sEVs) as a New Promising Aim of Research in the Immunopathology of CRS

Small extracellular vesicles (sEVs) are extracellular vesicles of endosomal origin. sEVs circulate in presumably all body fluids including blood, plasma, nasal lavage fluid, and bronchoalveolar fluid and are important mediators of cell to cell communication. They transport complex cargo components, i.e., proteins, lipids, and nucleic acids and sEVs play a role in various physiological processes, but importantly have immunomodulatory functions and are able to shift the Th1-Th2 balance [155]. Proteomic analysis of sEVs isolated from nasal lavage fluid of CRSwNP reported significantly different content in comparison to controls and revealed potential disease biomarkers such as cystatin, peroxiredoxin-5, and glycoprotein VI [156] or in another study the protease inhibitor cystatin-2 [157]. Additionally, sEVs are enriched in pappalysin and serpins potentially involved in NP formation [158,159]. Thus, sEVs modulate a wide spectrum of functions and their presence in all body fluids is of great value to potential diagnostic and therapeutic solutions in CRS.

## 4. Summary

Sinusal AE forms a structural and functional barrier that modulates proper interaction of the host microenvironment, the microbiome, EFs, and the immune response. In the course of CRS, the epithelial barrier remains impaired. Bacteria, fungi, viruses, allergens, and air pollution stimulate the AE that secretes cytokines, leading to activation of IIS including ILCs, neutrophils, monocytes, basophils, eosinophils, NK cells, and DCs. The antigen presentation of DCs leads to the polarization of T lymphocytes which invoke AIR. An imbalance between the physiological microbiome in sinonasal cavity and those in the course of CRS might orchestrate inflammation [90]. Tissue remodeling is among the most relevant aspects of CRS pathogenesis. Changes in the sinusal AE mainly include dysfunctions in the cilia and TJs, fibrosis, GC hyperplasia and BMT, NP formation, angiogenesis, and osteitis [160,161]. Although various widely known agents contribute to CRS, the molecular bases of disease still remain elusive and need further investigation.

## Data Availability

Not applicable.

## Abbreviations

AE	airway epithelium
AERD	aspirin exacerbated respiratory disease
AIR	adaptive immune response
BMT	basement membrane thickening
CIRP	cold-inducible RNA binding protein
CIS	complement immune system
CRS	chronic rhinosinusitis
CRSsNP	chronic rhinosinusitis without nasal polyps
CRSwNP	chronic rhinosinusitis with nasal polyps
CTGF	connective tissue growth factor
DCs	dendritic cells
DUOX	dual oxidase
ECM	extracellular matrix
EFs	environmental factors
EGR2	early growth response 2
EMT	epithelial to mesenchymal transition
GCs	goblet cells
HSP47	heat shock protein 47
HIF-1α	hypoxia-inducible factor 1α
HMGB1	high mobility group box 1 protein
IFN-γ	interferon-γ
IIS	innate immune system
IL	interleukin
ILCs	innate lymphoid cells
JNK	c-Jun N-terminal kinases
MAPK	mitogen-activated protein kinase
MCs	mast cells
miRNAs	microRNAs
MMP	metalloproteinase
NADPH	nicotinamide adenine dinucleotide phosphate
NF-κβ	nuclear factor κβ
NKs	natural killer cells
NO	nitric oxide
NOX4	NADPH oxidase 4
NPs	nasal polyps
OX40L	OX40 ligand
PAMPs	pathogen associated molecular patterns
PCI	persistent chronic inflammation
PD-1	programmed cell death 1
PPAR-γ	peroxisome proliferator-activated receptor γ
RAGE	receptor for advanced glycation end products
rhHMGB1	recombinant human high mobility group box 1 protein
ROS	reactive oxygen species
SCCs	solitary chemosensory cells
sEVs	small extracellular vesicles
SM	sinus mucosa
T2R38	taste receptor 2 member 38
T2Rs	taste receptors
TGFβ	transforming growth factor β
TIMP-2	tissue inhibitor of metalloproteinase-2
TJs	tight junctions
TLRs	Toll-like receptors
TNF-α	tumor necrosis factor α
Treg	T regulatory cell
TSLP	thymic stromal lymphopoietin
VEGF-A	vascular endothelial growth factor A
ZO-1	zonula occludens
